# Estimating relationships between phenotypes and subjects drawn from admixed families

**DOI:** 10.1186/s12919-016-0056-3

**Published:** 2016-10-18

**Authors:** Elizabeth M. Blue, Lisa A. Brown, Matthew P. Conomos, Jennifer L. Kirk, Alejandro Q. Nato, Alice B. Popejoy, Jesse Raffa, John Ranola, Ellen M. Wijsman, Timothy Thornton

**Affiliations:** 1 Division of Medical Genetics, Department of Medicine, University of Washington, Seattle, WA 98195 USA; 2 Department of Biostatistics, University of Washington, Seattle, WA 98195 USA; 3 Department of Statistics, University of Washington, Seattle, WA 98195 USA; 4 Institute for Public Health Genetics, University of Washington, Seattle, WA 98195 USA

## Abstract

**Background:**

Estimating relationships among subjects in a sample, within family structures or caused by population substructure, is complicated in admixed populations. Inaccurate allele frequencies can bias both kinship estimates and tests for association between subjects and a phenotype. We analyzed the simulated and real family data from Genetic Analysis Workshop 19, and were aware of the simulation model.

**Results:**

We found that kinship estimation is more accurate when marker data include common variants whose frequencies are less variable across populations. Estimates of heritability and association vary with age for longitudinally measured traits. Accounting for local ancestry identified different true associations than those identified by a traditional approach. Principal components aid kinship estimation and tests for association, but their utility is influenced by the frequency of the markers used to generate them.

**Conclusions:**

Admixed families can provide a powerful resource for detecting disease loci, as well as analytical challenges. Allele frequencies, although difficult to adequately estimate in admixed populations, have a strong impact on the estimation of kinship, ancestry, and association with phenotypes. Approaches that acknowledge population structure in admixed families outperform those which ignore it.

## Background

Estimates of the kinship coefficient, defined as the probability that 2 alleles randomly sampled from 2 subjects are identical by descent, have many uses [1]. These include verifying pedigrees and sample identity [2], and tests for association [3]. Pruning markers for linkage disequilibrium (LD) [3] and minor allele frequency (MAF) improves kinship estimation. Sequence data offers additional challenges by discovering novel and very rare variants without accurate MAFs. We investigate kinship estimators and subsets of whole genome sequence (WGS) data from Genetic Analysis Workshop 19 (GAW19) to examine bias and accuracy.

We explored whether genetic associations with systolic blood pressure (SBP) changed over time using longitudinal data. We estimated heritability and performed a kinship-adjusted half-genome-wide association test at the first and third visits for real SBP. We discuss the similarities, differences, and potential foundations for those changes.

Through both selection and drift, different populations can have different variants influencing the same trait, or very different frequencies of shared risk alleles. Admixture mapping takes advantage of these differences to identify risk loci [4]. Using the simulated SBP phenotype, we compare the power of RFMix, an accurate admixture mapping approach [4], to a traditional association test.

When testing for association between genotype and phenotype, principal components (PCs) are often included as covariates to minimize the effects of population structure. We investigated how well PCs estimated on different subsets of the data were able to capture ancestry proportions. Performance was evaluated with the coefficient of determination.

## Methods

### Genetic map and markers

We used GAW19 genome-wide association study (GWAS) data for odd-numbered autosomes for association testing and admixture mapping. Data were available on 959 subjects from 20 pedigrees. Sex-averaged map positions (cM) were converted by the Haldane map function from the Rutgers framework map with all of the Single Nucleotide Polymorphism database 134 (dbSNP134) variants [5]. The GAW19 WGS data for odd-numbered autosomes for 464 subjects from 20 pedigrees that passed the Support Vector Machines (SVM) filter and were missing 10 % or less data were extracted with VCFtools [6]. Sex-averaged positions (cM) were linearly interpolated for the WGS data using the GWAS markers as a framework panel. We extracted exomes from WGS data using the 1000genomes Phase3-like BED file (http://www.1000genomes.org/category/exome).

### Kinship estimation

We pruned WGS variants for LD in PLINK [7] (r^2^ ≤ 0.2), applied filters described below, and pruned for LD with SNPRelate [8] (r^2^ ≤ 0.1). The Agnostic design includes every 100th variant: 21,484 WGS variants. Agnostic variants are rare: 58 % have founder MAF of 1 % or less. The Selective design includes variants with MAF 5 % or greater: 64,389 WGS and 7215 exome variants. With an allele frequency spectrum comparable to the Selective design, our Homogenizing design includes the 30,710 WGS variants with alternate allele frequencies that minimally vary across the African (AFR), Native American (AMR), Asian (ASN), and European (EUR) populations [9] (maximum difference/overall frequency ≤2). This is similar to an approach to reduce bias caused by population structure [10].

**Fig. 1:**
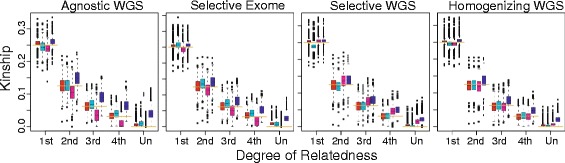
Box-plot comparison of pairwise kinship estimates from WGS vs. exome data. Blue, PLINK method of moments; cyan, PC-Relate; magenta, KING-robust; red, maximum likelihood; Un, unrelated; WGS, whole genome sequence

Within SNPRelate, we applied three estimators: method of moments (MoM [7]), maximum likelihood (MLE [1, 3]) for non-inbred pairs, and robust Kinship-based INference for Genome-wide association studies (KING [2]). No monomorphic single-nucleotide polymorphisms (SNPs) were evaluated and MAFs were estimated from the sample. The fourth estimator, PC-Relate [11], is a moment estimator that adjusts for population structure using PCs, estimated here from the GWAS data using PC-AiR [11] with the pedigree-based kinship values. We report the weighted average of chromosome-specific estimates, with negative values set to zero. We evaluate how often each estimator would assign pairs of subjects to their pedigree-based relationship by rounding to the nearest expected value of kinship for unrelated pairs, first-, second-, third-, or fourth-degree relatives in an outbred pedigree (represented by gold bars in Fig. 1).

### Association testing and longitudinal analysis of systolic blood pressure data—real data

We examined the role age may play on genetic effects on SBP by fitting a linear mixed-effects model to the longitudinal data for 916 individuals and 2189 SBP observations. Using the pedigree-based kinship matrix, we estimated the additive variance as a function of cubic splines of age, with log of the environmental variance similarly fit. SBP was adjusted using fixed effects for medication use, smoking status and gender, as well as smoking status and gender-specific curves for age.

We performed a half-genome scan for association with adjusted SBP at the first and third visits using Efficient Mixed-Model Association eXpedited (EMMAX) [12]. EMMAX is a mixed-model approach that calculates and uses an empirical genetic relatedness matrix (GRM) to account for both relatedness and population structure with a variance component for additive polygenic effects. A conservative Bonferroni correction for the association tests is 1.05 × 10^−7^ (0.05/472,049 markers).

### Association testing—simulated data

Admixture and association analysis of the simulated SBP at the first visit, adjusted as described above, included 955 subjects with GWAS data. Local ancestry was estimated by RFMix [4]. We phased the samples and imputed missing genotypes using Beagle3 [13] with 112 European (CEU [Northern Europeans from Utah]), 147 Yoruban (YRI [Yoruba in Ibadan, Nigeria]) and 63 Human Genome Diversity Project (HGDP) Native American (AMR [Admixed American]) samples [14, 15]. We included the 242,566 markers shared by GAW19 and reference panels. Proportion of ancestry from reference populations is an average of local ancestry values. Relatedness among subjects was included as a kinship matrix, φ, estimated by PC-Relate so as to adjust for the proportion of total ancestry from each reference population. We fit a linear mixed model at each marker, assuming the trait is Normally distributed with a mean equal to an intercept plus a main effect for ancestry and variance φσ_g_ + *I*σ_e_, where *I* is the identity matrix. Ancestry for CEU, YRI, and AMR were each fit separately. For admixture mapping, we use twice the whole-genome-wide nominal *p* value of 7 × 10^−6^, which has previously shown a type I error of 0.05 [16]. For association mapping, we fit the same model using allelic SNP dosage as the predictor and apply the same Bonferroni threshold as for the analysis of real data, 1.05 × 10^−7^.

### Population structure

We began with the WGS data for 102 unrelated subjects from the GAW19 pedigrees. We created four subsets of variants based upon their MAF: rare (MAF <0.01 or <0.05) and common (MAF >0.01 or >0.05). There were 7,407,452 SNPs (MAF <0.05 = 5,803,244 SNPs; MAF <0.01 = 4,522,880 SNPs). We estimated R^2^, the coefficient of determination, from a linear regression model with 10 PCs from a PC analysis (PCA) as predictors and CEU, AMR, and YRI ancestry proportions from a supervised ADMIXTURE [17] analysis as the response. Details on the supervised ADMIXTURE analysis are described elsewhere [18]. We performed a PCA with a GRM, Ψ, with (*i, j*)^th^ entry

1 $$ {\psi}_{ij}=\frac{{\displaystyle \sum_{s=1}^S\left(\left({\displaystyle {G}_i^s}-2{\widehat{p}}_s\right)\left({\displaystyle {G}_j^s}-2{\widehat{p}}_s\right)\right)}}{{\displaystyle \sum_{s=1}^S2{\widehat{p}}_s\left(1-{\widehat{p}}_s\right)}} $$

where *S* is the number of variants, *G*_*i*_^*s*^ and *G*_*i*_^*s*^ are the number of minor alleles (0, 1, or 2) that individuals *i* and *j*have at marker *s*, and *p*_s_ is the MAF at marker *s*. Unlike the EIGENSTRAT method [19], the entries in this GRM are calculated using ratios of sums, so low-frequency variants do not distort results.

## Results

### Kinship estimation

The MoM approach and Agnostic design provide the least-reliable kinship estimates (see Fig. 1), whereas the MLE values were consistently accurate. WGS and exome data performed comparably within the Selective design, whereas the Homogenizing design appeared to be more precise. MLE, KING, and PC-Relate performed similarly under the Homogenizing design. Excluding the Agnostic approach, the MLE and PC-Relate were each able to correctly assign relationships for more than 90 % of pairs of first- and second-degree relatives and unrelated subjects (Table 1). Distant relationships cannot be reliably determined by any method.

**Table 1:** Rate of successfully classified relationships. Frequency pairs within each relationship are correctly assigned to this degree of relationship

		Degree of relationship				
Design	Estimator	1st	2nd	3rd	4th	Unrelated
Agnostic WGS	MLE	99.7 %	91.0 %	76.1 %	60.2 %	99.7 %
	MoM	100 %	93.4 %	54.4 %	16.9 %	14.5 %
	KING	98.9 %	65.2 %	30.5 %	14.9 %	100 %
	PC-Relate	99.7 %	94.1 %	81.9 %	57.9 %	93.9 %
Selective WGS	MLE	99.7 %	92.7 %	76.3 %	61.3 %	99.9 %
	MoM	100 %	97.4 %	78.4 %	38.1 %	58.0 %
	KING	100 %	96.7 %	80.9 %	47.3 %	86.3 %
	PC-Relate	99.5 %	91.8 %	78.4 %	62.6 %	100 %
Selective exome	MLE	99.7 %	93.2 %	80.2 %	62.6 %	99.2 %
	MoM	99.7 %	96.5 %	71.6 %	33.0 %	43.4 %
	KING	99.5 %	80.0 %	52.4 %	30.5 %	99.5 %
	PC-Relate	99.7 %	96.5 %	79.8 %	52.4 %	97.1 %
Homogenizing WGS	MLE	99.5 %	91.0 %	74.2 %	57.3 %	100 %
	MoM	99.5 %	96.3 %	79.6 %	44.9 %	61.7 %
	KING	99.5 %	88.1 %	73.3 %	59.5 %	99.9 %
	PC-Relate	99.7 %	92.7 %	79.6 %	62.0 %	100 %

### Association testing and longitudinal analysis of systolic blood pressure data—real data

**Fig. 2:**
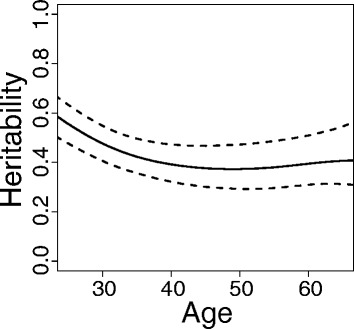
Heritability estimate from longitudinal analysis as a function of age. Mean baseline age was 39.0 years (SD = 16.3) with a median follow-up of 10.4 years (Q1-Q3: 1.8–14.4). Number of SBP observations in each age (in years) strata: <30: 483, [30,40): 415,[40,50): 438, [50,60): 326, ≥60: 324. The upper/lower dotted lines represent 95 % confidence bands

Environmental variance generally increased as a function of age, shown in the narrow-sense heritability estimates presented in Fig. 2. In those subjects who also had genotype data (*N* = 831; 2060 SBP measurements), we found the heritability estimates were robust to kinship estimators (KING, PC-Relate, and pedigree-based). The adjusted first- and third-visit SBP values are quite different, with a correlation of 0.48, and the heritability estimate from EMMAX for the first-visit SBP is 0.26 and 0.13 for the third-visit SBP.

**Fig. 3:**
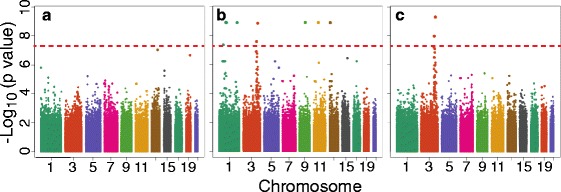
EMMAX association test results for real first (**a**) and third (**b**) visit adjusted SBP, and third visit excluding an outlier (**c**). Bonferroni-corrected significance threshold is shown with a horizontal line

The EMMAX approach found no significant associations with SBP at the first visit (Fig. 3a), but identified several for the third visit (Fig. 3b). Although there was some inflation of the test statistics for the third visit (genomic control inflation factor, λ = 1.03), this was not seen for the first visit (λ = 1.002). The string of extreme *p* values in Fig. 3b could be caused by an outlier. We repeated EMMAX analyses without the subject with the most extreme SBP at the third visit (Fig. 3c). Although λ did not change much (λ = 1.034), the association signals on chromosomes 1, 9, 11, and 13 were eliminated. The remaining signal on chromosome (chr) three remained, where the top SNP (rs7637973, in the *LRRC31* gene) had a *p* value of 5 × 10^−10^.

### Association testing—simulated data

**Fig. 4:**
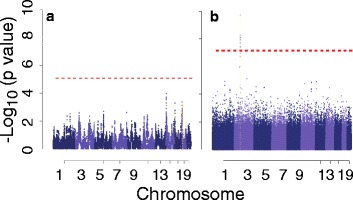
Admixture mapping (**a**) and association testing (**b**) for adjusted simulated SBP using local ancestry. Significance thresholds are shown with a red horizontal line, based on theory [16] (**a**) or Bonferroni correction (**b**)

Admixture mapping had no power at the significance level of 1.4 × 10^−5^. If we reduce the significance threshold to 5 × 10^−4^, we have 17 % power across 200 simulated replicates to detect the variant in the *P2RX5* gene on chr17 and 81 % power to detect the variant in the *COL5A3* gene on chr19. For association mapping, we observed 100 % power across 200 replicates to detect the causal variant in the *MAP4* gene on chr3 at the significance level of 10^−7^. Figure 4 shows the distribution of *p* values for each approach for the first simulated replicate.

### Population structure

As shown in Table 2, the MAF of marker data influenced our ability to capture population substructure. PCA with rare variants captured the YRI structure substantially better than using common variants, whereas common variants captured CEU/AMR ancestry better than the rare variants. The boundary between common and rare variation (1 % vs. 5 % MAF) had relatively minor influence on results.

**Table 2:** Principal components analysis with rare and common WGS variants

			PCA-Seq			EIGENSTRAT		
	PCs	MAF	CEU	AMR	YRI	CEU	AMR	YRI
R^2^ for rare variants	10	<0.01	0.18	0.16	0.33	0.17	0.15	0.33
		<0.05	0.79	0.80	0.55	0.78	0.79	0.54
	20	<0.01	0.42	0.42	0.43	0.42	0.42	0.42
		<0.05	0.83	0.84	0.66	0.82	0.83	0.65
R^2^ for common variants	10	<0.01	0.95	0.95	0.13	0.95	0.95	0.17
		<0.05	0.95	0.95	0.13	0.95	0.95	0.11
	20	<0.01	0.97	0.97	0.22	0.97	0.97	0.41
		<0.05	0.97	0.97	0.22	0.97	0.97	0.23
R^2^ for all variants	10	—	0.95	0.96	0.18	0.96	0.96	0.22
	20	—	0.97	0.97	0.41	0.97	0.97	0.49

*MAF* minor allele frequency, R^2^, the coefficient of determination from a linear regression model with 10 PCs included as predictors and the proportion of CEU, AMR, and YRI ancestry proportions from ADMIXTURE analysis as the response

## Discussion

We have shown that the frequency of alleles included in kinship, association, and population structure estimation have strong influences on their results. Kinship estimation is most accurate when markers are restricted to common variants that are not ancestry informative, and the moment estimator showing the least bias incorporated ancestry-informative PCs. Admixture mapping and association testing each identified different causal genes for the simulated adjusted SBP, likely a consequence of differences in frequency of risk alleles in the AMR and CEU reference populations. This warrants a future analysis evaluating a single test of ancestry at each locus using a 2-degrees-of-freedom test, as opposed to treating each ancestry separately. When estimating population structure, the marker data must be selected using the frequency of alleles in multiple relevant reference populations in order to adequately capture the complexity of ancestry in admixed populations.

Association testing found little evidence for real adjusted SBP loci, and the inflation of EMMAX results with third-visit SBP was likely caused by the increase in environmental variance in the trait as age increased, consistent with the change in heritability estimates over time. There could be shared environmental factors acting on third-visit SBP that are not being modeled. This would cause the systematic inflation of test statistics across the genome.

## Conclusions

Variants with imprecise allele frequencies bias estimates of kinship, PCA, and association testing. Admixture mapping and association testing proved complementary. The influence of environment on estimates of heritability and association appear to have been revealed by analysis of longitudinal data.
